# Evolution and Virulence of Influenza A Virus Protein PB1-F2

**DOI:** 10.3390/ijms19010096

**Published:** 2017-12-29

**Authors:** Ram P. Kamal, Irina V. Alymova, Ian A. York

**Affiliations:** 1 Battelle Memorial Institute, Atlanta, GA 30329, USA; 2 Influenza Division, National Center for Immunization and Respiratory Diseases, Centers for Disease Control and Prevention, Atlanta, GA 30333, USA; xeq3@cdc.gov (I.V.A.); ite1@cdc.gov (I.A.Y.)

**Keywords:** influenza, PB1-F2, virulence, pathogenesis

## Abstract

PB1-F2 is an accessory protein of most human, avian, swine, equine, and canine influenza A viruses (IAVs). Although it is dispensable for virus replication and growth, it plays significant roles in pathogenesis by interfering with the host innate immune response, inducing death in immune and epithelial cells, altering inflammatory responses, and promoting secondary bacterial pneumonia. The effects of PB1-F2 differ between virus strains and host species. This can at least partially be explained by the presence of multiple PB1-F2 sequence variants, including premature stop codons that lead to the expression of truncated PB1-F2 proteins of different lengths and specific virulence-associated residues that enhance susceptibility to bacterial superinfection. Although there has been a tendency for human seasonal IAV to gradually reduce the number of virulence-associated residues, zoonotic IAVs contain a reservoir of PB1-F2 proteins with full length, virulence-associated sequences. Here, we review the molecular mechanisms by which PB1-F2 may affect influenza virulence, and factors associated with the evolution and selection of this protein.

## 1. Introduction

Influenza A viruses (IAVs) cause major human health and economic burdens through both seasonal epidemics and occasional pandemics. The IAV genome is segmented, with each of its eight segments encoding one or more proteins. In addition to ten classical influenza proteins (PB1, PB2, NP, HA, PA, NA, M1, M2, NS1, and NS2), influenza viruses also encode a number of proteins via frame-shifts and complementary sequences [1]. These accessory proteins are often more variable than the classical ones in their sequence and expression, and in many cases are not required for virus replication and transmission. However, their expression might enhance influenza virulence [2,3,4]. These proteins are therefore of a great interest as virulence factors that may help identify influenza strains and clades that have immense pathogenic potential and that are of a significant epidemiological concern.

One of the most interesting and puzzling of these accessory proteins is PB1-F2. This protein is encoded by a +1 alternate open reading frame (ORF) in the PB1 segment, and was discovered while looking at putative CD8+ T cell epitopes encoded by alternative reading frames of influenza gene segments of mouse-adapted A(H1N1) laboratory strain A/Puerto Rico/8/34 (PR8) [5]. The other accessory protein encoded by PB1 gene segment is N40 [6]. PB1-F2 was shown to be widely expressed by many (but not all) IAV strains [5]. Of note, the PB1-F2 ORF was not identified within the influenza B viruses [5]. The presence or absence of PB1-F2 as well as its sequence and length has been linked to viral virulence. Unsurprisingly, the details of its contribution to virulence are strain, cell-type, and host specific [7,8,9,10,11]. In this review we discuss the findings indicating that PB1-F2 is an important factor of influenza virulence that should be a regular component of virus monitoring.

## 2. Structure

**Figure 1:**
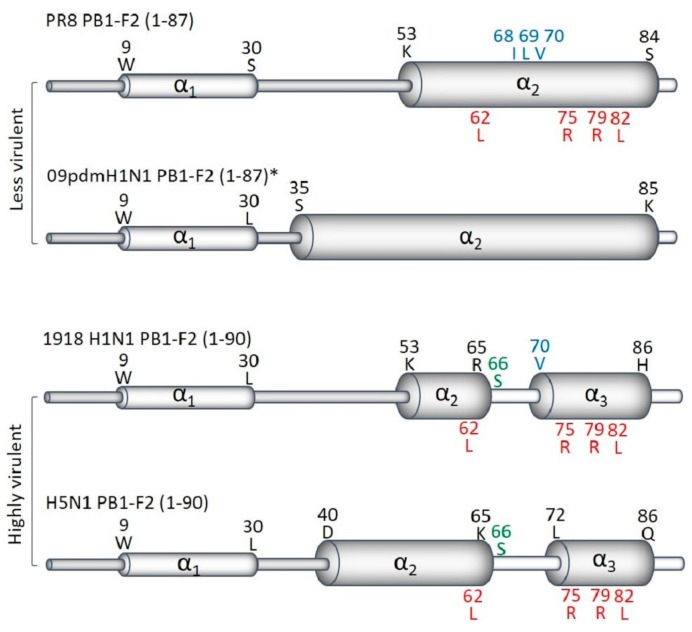
Secondary structure of PB1-F2 C-terminus differ between highly and less virulent viruses. (Adapted from Solbak et al., 2013 [16]). The overall predicted secondary structures of full-length PB1-F2 of PR8, 2009pdmH1N1 *, 1918 H1N1 and HPAIV H5N1 are shown. Regions with the best-defined α-helical structure in the C-terminal are denoted with broad cylinders, whereas regions with lesser-defined α-helical structure in the N-terminals appear as thin cylinders. PB1-F2 of less virulent strains have one longer α-helix, while the highly virulent viruses contain two α-helices in its C-terminus. The positions and residues of virulent sequence variants of PB1-F2 are shown in green (N66S), blue (the cytotoxic motif), and red (the inflammatory motif). * All 2009pdmH1N1 express PB1-F2 of only 11 amino acids. The structure shown was predicted for full-length PB1-F2, restored by mutating pre-mature stops codons.

PB1-F2 is a small protein, with the PR8 version being 87 amino acids long with a molecular weight of 10.5 kDa [5]. Structural characterization of the PR8 PB1-F2 revealed that, depending on solvent conditions, it is capable of forming a random coil or a secondary α-helical or oligomeric β-sheet enriched structure [12,13]. PB1-F2 targets cellular membranes, including mitochondrial [14,15], and both secondary α-helical and β-sheet PB1-F2 structures were formed in membrane-mimicking environments. Two types of helix structures have been described (Figure 1). The less virulent PB1-F2 protein from PR8, and a full-length protein reconstructed from the 2009 A(H1N1)pdm09 pandemic virus, A/California/04/2009, by eliminating premature stop codons, formed a continuous C-terminal α-helix [16] (Figure 1). In contrast, PB1-F2 from the highly pathogenic pandemic A(H1N1) A/Brevig Mission/1/1918 (1918 H1N1) and avian A(H5N1) A/duck/Guangdong/12/2000 viruses [16], adopts two short helix-like regions between residues 9 and 20 at the N-terminus (~40 amino acids) and an extended α-helix of residues 55–85 at the C-terminus (~36 amino acids), which are connected by a coil-like region. It has been suggested that a divided C-terminus may be a structural signature of highly pathogenic IAV strains. However, more examples need to be tested to confirm this proposition. While the C-domain anchors the molecule in membranes, the mainly unstructured N-domain remains free in solution.

The ability of protein to form β-sheets [12] and (as a result) insoluble oligomeric amyloid fibers on the membranes of infected cells [13,17,18] is likely to be sequence-specific, because PB1-F2 from different IAV strains have varying abilities to adopt β–sheets. Several basic amino acid sets have been predicted to contribute to this phenomenon with the strongest being the C-terminal 68-ILVFL-72 motif [12].

## 3. Expression

**Figure 2:**
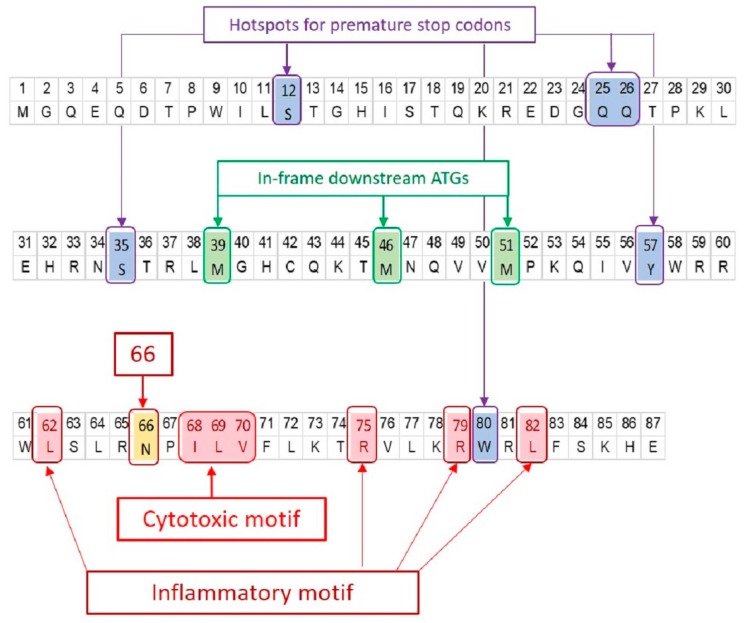
Signature positions on PB1-F2 protein. The amino acid sequence of PR8 PB1-F2 is shown. The significant positions of interest found in PB1-F2 of all IAVs are marked irrespective of their presence in PR8 PB1-F2. PB1-F2 ORF has three in-frame downstream translation initiation sites (**highlighted green**). Many IAV isolates express truncated PB1-F2 due to the presence of premature stop codon(s) (**highlighted blue**). Three major sequence variants of PB1-F2 i.e., N66S (**highlighted yellow**), cytotoxic motif (**highlighted red**) and inflammatory motif (**highlighted red**) have been associated with enhanced virulence.

PB1-F2 is translated from the 4th start codon in the PB1 gene segment through leaky ribosomal scanning, which is favored by an optimal Kozak sequence at start codon of PB1-F2, while the start codon for PB1 and the 2nd and 3rd potential start codons all have sub-optimal Kozak sequence [5]. In the initial description of PB1-F2, 64 of the 75 publically available influenza A PB1 sequences were shown to include full-length PB1-F2 proteins of 87–90 amino acids [5]. All “older” pandemic 1918, 1957, and 1968 IAVs contained a full-length PB1-F2. It was later shown that a number of IAV possess truncated PB1-F2 with one or more premature stop codons [4,19,20] or lacking an initiating ATG [5]. The expression of PB1, PB1-F2 and N40 (a third protein expressed from the PB1 gene segment [6]) have been shown to be interdependent [6,21]. Ablation of the PB1-F2 start codon, but not truncation at codon 12, enhances N40 expression and hampers virus replication [6,21] Premature stop codons may be present in several PB1-F2 ORF positions including 12, 25, 26, 38 and 58 (PB1-F2 numbering), individually or in combination (Figure 2). For example, the 2009 A(H1N1)pdm09 virus strain A/California/04/2009 does not express PB1-F2 due to the presence of three stop codons in the PB1-F2 ORF at positions 12, 58, and 88 [9]. Even so, truncated PB1-F2 proteins (mainly C-terminal version) may be expressed from downstream ATGs (e.g., at position 39, 46, and 51) even in the presence of stop codons (Figure 2) [4,19] or in the absence of an initiation ATG [5]. Truncated proteins may retain some of PB1-F2 functions, especially those that are the attributes of its C-terminus, and therefore be as virulent as a full-length protein.

Viruses containing full-length PB1-F2 ORF (such as PR8) may express several different forms of the protein varying in their length [5]. The significance of these variants for protein virulence is unknown as yet, and their analysis is complicated by the fact that all forms of PB1-F2, and especially the truncated variants [5], are unstable and have short half-lives. Increasing PB1-F2 stability by mutating potentially ubiquitinated residues in the C terminus strengthened its polymerase enhancing and interferon (IFN) antagonistic functions [22].

The level of PB1-F2 expression was shown to vary among different IAV strains [22], host and cell types, and be regulated at the translational level by sequences within the PB1 gene segment [23]. Variations within these sequences can result in low levels of protein expression in spite of an apparently intact PB1-F2 ORF [23].

## 4. Molecular Mechanisms of Pathogenesis

At the molecular level, many activities have been reported for PB1-F2, most of them associated with the C-terminal region of the protein. Minor changes in PB1-F2 sequence can lead to substantial differences in functionality. The inconsistency of PB1-F2 activities between viral strains, different cell types, and hosts complicates the understanding of the role of PB1-F2 in actual infection. In addition, different localization patterns such as mitochondrial [5,24] or cytoplasmic or nuclear [7,25,26]) suggest that there may be multiple PB1-F2 activities. Molecular functions of PB1-F2 that have been identified so far include down-regulation of antiviral innate immunity, promotion of pro-inflammatory cytokines, induction of cell death, and enhancement of viral polymerase activity. Connections between particular molecular functions or interactions to PB1-F2 virulence are not always clear.

### 4.1. Regulation of Antiviral Innate Immunity

Innate immune responses normally not only thwart virus spread by inducing an early antiviral state in infected and adjacent cells, but also shape local inflammatory and adaptive immune responses. Mitochondria play important role in antiviral innate immune response through the mitochondrial antiviral signaling (MAVS) protein, which is a component of the RIG-I antiviral pathway [27,28]. After sensing viral RNA, RIG-I triggers MAVS activation, which leads to activation of IRF3/7 and NF-kB that in turn lead to the expression of IFNs and pro-inflammatory cytokines, respectively [29]. MAVS also recruits NOD-like receptor (NLR) family pyrin domain-containing 3 (NLRP3) to mitochondria, resulting in activation of NLRP3 inflammasomes and production of the pro-inflammatory cytokines IL-1β and IL-18 [30]. The balanced activation of all these pathways, along with multiple others, is important for combating and resolving viral infection, repair of damaged tissues, and generating adaptive immune response.

**Figure 3:**
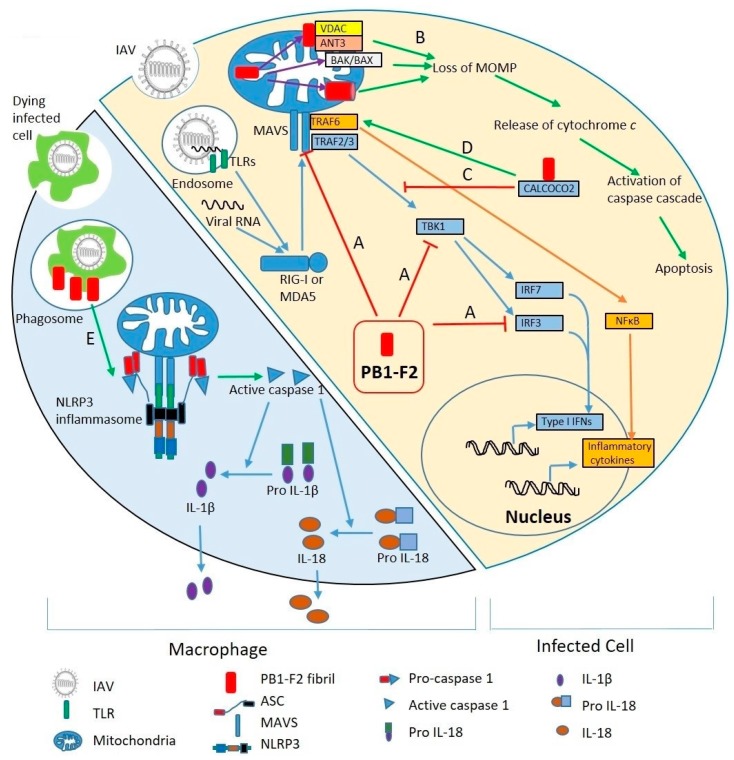
Molecular mechanisms of PB1-F2 virulence. Right side of the diagram shows an IAV infected cell; left side shows a macrophage phagocytosing virus-infected cells. (**Right**) Upon IAV infection, viral RNA is discovered by TLRs in endosomes and RIG-I and MDA5 in the cytosol. RIG-I and/or MDA5 triggers MAVS-mediated activation of the innate immune response. MAVS recruits TRAF3 and TRAF6. TRAF3 leads to production of Type I IFNs through IRF3 and IRF7 pathways. TRAF6, through NF-κB, stimulates expression of inflammatory cytokines. PB1-F2 inhibits type I IFNs by interfering with MAVS, TBK1 and IRF3 (A). PB1-F2 localized to mitochondria leads to loss of mitochondrial outer membrane potential (MOMP) (B) either by binding to mitochondrial membrane proteins VDAC and ANT3, activation of BAK/BAX, or pore forming through PB1-F2 oligomerization. MOMP loss releases cytochrome *c* into the cytosol, activates caspase cascades, and leads to apoptosis. By binding to CALCOCO2 (C), PB1-F2 inhibits IFN signaling pathway, but also stimulates TRAF6-mediated NF-κB activation, thus promoting inflammatory cytokine production (D). (**Left**) PB1-F2 stimulates NLRP3 inflammasomes in macrophages upon phagocytosis of virus-infected dead cells (E). Inflammasome activation leads to maturation and release of IL-1β and IL-18.

PB1-F2 has been shown to interfere with these regulatory mechanisms (Figure 3) through direct interaction with MAVS and other components of the RIG-I/MAVS system [31,32,33,34,35,36], which has been linked to changes in cytokine expression in several experimental systems. For example, macrophages infected with PB1-F2-expressing virus produced less IFN-β [34,37,38], IL-6, IL-8 [39], and/or IL-1β [25] compared to those infected with the same viruses lacking PB1-F2.

In addition to inhibition of IFNs through interference with IRF3 pathway, PB1-F2 promotes TRAF6 mediated activation of NF-kB resulting in enhanced expression of pro-inflammatory cytokine (Figure 3) including IFN-β [34], IL-1β [9,40,41], GM-CSF, IFN-γ, IL-6 and IL-8 [9,41,42]. PB1-F2 interaction downstream of RIG-I/MAVS, i.e., with the IRF3 [38], the NF-κB [43], and TBK1 [36] pathways have also been described. PB1-F2 interactions with IRF3 and TBK1 inhibit IFN signaling pathways. Similarly, the NF-κB pathway is inhibited by PB1-F2’s interaction with IκB kinase β (IKKβ) [43]. PB1-F2 interaction with CALCOCO2 inhibits TRAF2/3 mediated IFNs and promotes TRAF6 mediated-inflammatory cytokines. [36] Translocation of PB1-F2 into mitochondria leads to a loss of membrane potential and formation of abnormal fragmented mitochondria that provide signals for activation of NLRP3 inflammasomes and thus pro-inflammatory cytokines [27,30,33,40,44].

Thus PB1-F2 has the capability of both inhibiting antiviral cytokines and enhancing expression of inflammatory cytokines (Figure 3). Animal studies suggest that pro-inflammatory effects may be important relatively late in infections, but inhibition of the very early inflammatory response may also be an important component of pathogenesis.

### 4.2. Enhancement of Viral Polymerase Activity

As with several other PB1-F2 functions, its effect on polymerase function is not universal and varies among different IAV strains. Moreover, increased polymerase activity does not always correlate with higher viral titers. PB1-F2 from PR8 virus was shown to co-localize with the PB1 polymerase in epithelial cells, resulting in increased PB1 retention in the nucleus and enhanced polymerase activity [26], an effect that was mediated through the N-terminus of PB1-F2 [45]. The increased polymerase activity was associated with larger plaques [26] and increased viral titers in tissue cell cultures [7,26]. Most subsequent studies find little or no effect of PB1-F2 on viral replication either in cultured cells [4,5,9,24,34,39,46] or in infected mice lungs [4,24,35,47,48]. Since many natural isolates of IAV (e.g., virtually all A(H1N1)pdm09 strains) completely lack an ORF for PB1-F2 due to multiple stop codons, it is clear that PB1-F2 is not required for IAV replication. Besides PB1-F2 length and expression level, this function may be affected by N40 expression levels [49,50].

### 4.3. Induction of Cell Death

The first described function of PB1-F2 was induction of apoptotic cell death in immune cells (Chen et al., 2001 [5]) and has been confirmed by multiple studies since then [5,7,37,51]. Not all cell types are affected by PB1-F2-induced apoptosis [5,24,34]. Macrophages [8,37,39,51] and monocytic U937 or RAW264.7 cells [5,7,11] appear to be the most sensitive to PB1-F2 priming. As well, cell death has been associated with PB1-F2 from some viral strains, such as H1N1 [5,7,15,20,24,33], but not from others such as A(H3N2) and A(H5N1) [7,11,19].

Induction of apoptotic cell death is mainly attributed to the mitochondrial localization of PB1-F2 from some IAVs. Mitochondrial localization of PB1-F2 is achieved by its mitochondrial targeting sequence, a short arginine-rich motif spanning minimum of amino acids 65–75 [12,14,15]. PB1-F2 translocates into mitochondria via the Tom40 channel [33], a main component of the outer membrane translocation machinery for mitochondrial proteins, and leads to permeabilization and destabilization of the outer mitochondrial membrane, leading to macromolecular leakage and apoptosis [52,53]. Among the mechanisms to be involved in the mitochondrial membrane permeabilization by the PB1-F2 are formation of pores due to accumulation of amyloid fibers [13,17,18], PB1-F2 binding to the voltage-dependent anion channel 1 (VDAC1) and adenine nucleotide translocator 3 (ANT3) proteins in the outer and inner mitochondrial membranes (respectively) [53], and activation of Drp-1- [33] and Bak/Bax- [11] pathways. Mitochondria-driven apoptosis is dependent on activation of caspase 1/3 pathways. Caspase-3-independent apoptosis has also been reported to be activated by PB1-F2 in epithelial sulfatide-enriched COS-1 cells [54].

In addition to apoptotic cell death, PB1-F2 possessing I68, L69, and V70 residues can trigger cytotoxic death in epithelial and immune cells [24]. PR8 mutants whose PB1-F2 lacked the PKC phosphorylation sites 27T and 35S showed reduced replication and apoptosis in primary human monocytes [51].

## 5. Pathogenesis in Animals

The ability of PB1-F2 to alter influenza virulence has been studied in a variety of mammalian (such as mice, ferrets, and pigs) and avian (such as chickens, ducks, and turkeys) hosts. Studies have used isogenic viruses differing in their ability to express PB1-F2 protein due to the addition of stop codons and mutation of the initiating ATG, or differing in PB1-F2 sequence to identify virulence-associated residues. Synthetic peptides corresponding to various PB1-F2 sequences have also been used to identify virulence-associated residues. In general, the conclusions from the various approaches have been consistent and they indicate that the pathogenic effects of PB1-F2 depend on the specific IAV strain and host model.

### 5.1. PB1-F2 Deletion

Zamarin et al. [4] were the first to find that the presence of PB1-F2 enhance the virulence of A/WSN/1933(H1N1) (WSN/33) in mice. Despite similar mouse lung titers at earlier infection time points, the WSN/33 expressing PB1-F2 was cleared from the lungs more slowly compared with the virus without PB1-F2. Expression of PB1-F2 from WSN/33 was associated with elevated mortality due to increased neutrophil lung infiltration and immunopathology [3,35]. Increased lung immunopathology in mice was also seen with PR8 expressing PB1-F2, compared to PR8 variants lacking PB1-F2 expression [11,38]. Immunopathology was characterized by high numbers of neutrophils, macrophages, and T cells in bronchoalveolar lavage fluid as well as epithelial cell hypertrophy and hyperplasia, necrosis, and fibrin deposition [11,40,55]. In general, in mammals the expression of PB1-F2 seems to delay the initial immune response and increase the pulmonary inflammatory response during the late infection stage. The presence of PB1-F2, inhibits expression of genes involved in macrophage activation, NK cell recruitment and functions in mice [48]. However, it enhances the expression of genes involved in epithelial damage, lymphocyte apoptosis and inflammatory cytokines/chemokines for granulocyte recruitment [48]. As a result of imbalanced host immuno-inflammatory responses, expression of PB1-F2 was shown to promote secondary streptococci [55] and staphylococci [50] pneumonia in mice. The ability of IAVs to generate an excessive inflammation and promote secondary bacterial pneumonia was noted to be a hallmark of PB1-F2 from highly pathogenic A(H5N1) and 1918, 1957, and 1968 pandemic strains [11].

Interestingly, experiments with restoring PB1-F2 expression in H1N1pdm09 showed little effect on virus virulence and secondary bacterial pneumonia in mice and ferrets, although some immune response modulation was observed [9,46]. Similarly to mice and ferrets, restoring expression of the H1N1pdm09 PB1-F2 only moderately impacted viral replication, lung histopathology, and cytokine response in pigs [56]. It seems that viruses that are intrinsically highly virulent may be less dependent on PB1-F2 for their pathogenic effects, since deleting PB1-F2 from A(H5N1) strains did not alter their pathogenicity in mice [7,25].

Several studies noted that effects of PB1-F2 expression within the same virus varies depending on the host. For example, the full-length PB1-F2 of A/swine/Kansas/77778/2007(H1N1) was more virulent in swine than in mice [42]. In birds, the PB1-F2 expression has been linked to reduced rather than increased virulence. In chickens, expression of PB1-F2 diminished the virulence of highly pathogenic H5N1 due to reduced immune responses [10,57]. An H3N2 swine virus lacking PB1-F2 infected turkey poults more efficiently than virus expressing PB1-F2 causing extensive histopathological changes in the intestine, although PB1-F2 expression did not alter this virus’s pathogenicity in swine [8]. Similarly, the presence of a full-length PB1-F2 reduced virulence of an H9N2 avian influenza virus in chickens, while also prolonging virus shedding [57].

### 5.2. PB1-F2 Sequence Variants

As with expression of full-length protein, sequence variation in PB1-F2 has been associated with changes in disease severity. For example, substituting PB1-F2 from the highly virulent pandemic 1918 H1N1 strain for the natural PR8 sequence increased virulence in mice, increasing secondary bacterial pneumonia and immunopathology [55]. The effect of PB1-F2 sequence variants is mainly studied using two experimental approaches; isogenic reassortant viruses generated by site-directed mutagenesis and synthetic peptides. Experiments with synthetic peptides utilize all or part of the PB1-F2 protein added to tissue culture medium, or instilled into mouse lungs. In spite of the unnatural mode of expression and localization, it has been shown that pathogenic effects induced by such peptides are generally similar to the same sequences expressed in the context of a viral infection [24,58,59], making this a useful approach for specifically identifying effects associated with specific PB1-F2 sequences as well as for identifying general PB1-F2 effects. However, results from peptide experiments do not always correlate with virus infection [11] perhaps because peptide effects are usually seen with higher doses which are unlikely to be achieved during virus infection. 

These studies have identified many sequence variants affecting pathogenic effects of PB1-F2. Of these, the three most important are N66S, the inflammatory motif, and the cytotoxic motif (Figure 1 and Figure 2).

#### 5.2.1. N66S

Comparing highly virulent to less virulent strains of A(H5N1) IAV implicated a change from Asn to Ser at position 66 of PB1-F2 (N66S) as having pathogenic significance [60]. This mutation has been shown to increase virus replication in a cell type and strain-specific manner [9,25,31]). The presence of 66S in pandemic 1918 H1N1 [60], A/Hong Kong/156/1997(H5N1) [60,61], A/Viet Nam/1203/2004(H5N1) [25], and A/swine/Kansas/77778/2007(H1N1) [42] strains has consistently led to enhanced viral virulence in mice, with increased viral titers and immunopathology. Infection with IAV containing PB1-F2 with 66S were characterized by early inhibition of IFNs, increased levels of pro-inflammatory cytokines, and enhanced lung infiltration of immune cells, when compared to the isogenic viruses expressing 66N. Neurotropism was also observed in case of A/Viet Nam/1203/2004(H5N1) when its PB1-F2 contained 66S [25]. However, restoring PB1-F2 with 66S in H1N1pdm09 increased expression of pro-inflammatory cytokines both in mice and ferrets but did not altered the virulence (Hai et al., 2010 [9]).

The pathogenic effects of 66S were found to be host specific. Expression of 66S in PB1-F2 of A/Viet Nam/1203/2004(H5N1) increased virus virulence in mice, but not in ducks [25]. Similarly, 66S increased virulence of A/swine/Kansas/77778/2007(H1N1) in mice, but not in pigs [42].

In general, the 66S variant appears to lead to suppressed early induction of IFN-β and promote pro-inflammatory cytokine expression, leading to increased lung immunopathology and disease severity.

#### 5.2.2. Inflammatory Motif

Comparison of PB1-F2 sequences from influenza strains that have different effects on lung inflammation identified an “inflammatory motif”, comprised of L62, R75, R79, and L82 residues [58]. Viruses with these residues tend to cause more inflammatory infiltrates in mouse lungs following infection or administration of synthetic peptides [58,59] and enhance secondary bacterial pneumonia [58,59,62].

All pandemic 1918, 1957, and 1968 IAVs contain PB1-F2 with a full set of inflammatory residues. As IAVs have evolved in humans following the pandemic, this motif has been gradually lost (Section 6), which has been linked to a reduction of the inflammatory response to infection [59,63].

#### 5.2.3. Cytotoxic Motif

Another motif I68, L69, and V70 identified within PB1-F2 of some IAV strains was also shown to enhance lung immunopathology and promote development of secondary bacterial pneumonia in mice through enhanced cytotoxicity [24]. The cytotoxic residues were found to be among those increasing PB1-F2 protein stability [49], mitochondrial localization [24,49], and inhibitory effect on IFN-β expression [49].

#### 5.2.4. Other Sequence Variants

PB1-F2 sequence variants other than 66S, inflammatory (L62, R75, R79, and L82), and cytotoxic (I68, L69, and V70) have also been reported to affect virus pathogenicity. For example, mutations T51M, V56A, and E87G in A/VietNam/1203/04(H5N1) virus reduced pathogenicity in mallard ducks [64].

### 5.3. Summary of Pathogenic Effects

In spite of the complex PB1-F2 strain-, length- and sequence-dependent effects on virulence, a number of common underlying themes can be identified. PB1-F2-induced immunopathology is a common effect, with the presence of full length PB1-F2 with virulent residues being associated with increased cellular infiltration and increased cytokine expression [3,9,11,24,34,41,58,60,61]. The PB1-F2-induced immunopathology associates with modulation in the innate immune response, since mice with varying components of the adaptive immune system responded similarly [61]. However, some experiments have found no effect on the inflammatory response despite the presence of some of the virulent residues [34,39,55] or an inhibitory effect, with PB1-F2 delaying or inhibiting inflammatory responses [25,41,65]; strain and host species differences are probably involved in this variation. Since increased cytokine responses have been linked with severe disease outcomes following influenza infection in humans [66,67], the potential link between PB1-F2 and virulence in humans is supported by animal experiments.

One of the most clinically important effects of influenza infection is its ability to render patients more susceptible to subsequent secondary bacterial infections. The presence of full-length PB1-F2 with specific virulent residues associates with increased susceptibility to secondary bacterial pneumonia in mice [24,50,55]. The effect of PB1-F2 on secondary bacterial pneumonia seems to be distinct from direct effects of the virus, since PB1-F2 synthetic peptides increase susceptibility to bacteria as well as viruses [55,58].

## 6. Prevalence and Evolution of PB1-F2 Virulent Residues

Influenza viruses are predominately pathogens of wild waterfowl and other birds, in which influenza viruses of many subtypes constantly reassort, mutate, and are carried around the world. At intervals, avian influenza viruses jump into mammalian hosts, producing infections that may be transient or that may become pandemic or endemic in their new hosts. Mammalian species in which influenza viruses have become well established include bats, horses, dogs, swine, and humans. As influenza viruses evolve in their new hosts, they undergo adaptive changes that can help identify features involved in pathogenesis. We and several other groups have analyzed the changes relevant to PB1-F2 virulence in different virus subtypes and hosts (Table 1). Since specific residues in C-terminus of PB1-F2 protein have been associated with enhanced pathogenicity, tracking variations in these residues (either due to mutations of the residues or through truncations that lack the C-terminus) is important. 

The frequency of PB1-F2 truncations varies widely by viral subtype (Table 1). For example, PB1-F2 of human seasonal H1N1 viruses acquired a premature stop codon at position 58 in 1950, since then all of them had truncated (57aa) PB1-F2 until 2009 [20]. Following the 2009 pandemic, multiple stop codons truncated PB1-F2 in H1N1pdm09 viruses at position 11 [9]. In contrast, since 1968 most human H3N2 isolates have had full-length PB1-F2, with the exception of the 2010–2011 season when over 40% of isolates had truncated PB1-F2 [19].

Different hosts tend to have IAV with different frequencies of truncated PB1-F2. PB1-F2 from swine isolates of the H3N2 and H1N2 subtypes most often express PB1-F2 of 62 or more amino acids [68], but, about 35% of current swine H1N1 IAV express PB1-F2 of shorter than 62 amino acids (Table 1). Equine influenza viruses are of the H3N8 subtype, and almost invariably (98% of sequences in databases) have PB1-F2 of 62 amino acids or longer. Two influenza subtypes are endemic in dogs: an H3N8 lineage that originated from horses, and an H3N2 lineage of avian origin. In contrast to the equine influenza viruses, canine H3N8 frequently (35%) contain truncated PB1-F2, while about 95% of canine H3N2 viruses express full-length proteins (Table 1).

Strains of avian influenza viruses that sporadically infect humans, including H5N1 high-pathogenic influenza viruses and H7N9 viruses, are most closely monitored through active surveillance and clinical isolates. In contrast to mammalian PB1-F2, avian IAV isolates typically express full length PB1-F2 of 87aa or longer [19]. However, the fraction of avian influenza virus isolates expressing truncated versions of PB1-F2 has increased following 2012, with over 60% of sequenced H5N1 containing truncated versions by 2013 [19].

The residues most clearly associated with PB1-F2 virulence are 66S, and the inflammatory L62, R75, R79, and L82 and cytotoxic I68, L69, and V70 motifs. The number of residues associated with virulence changes as influenza strains adapt to their mammalian hosts. The 1918 pandemic H1N1 [69], 1957 H2N2, and 1968 H3N2 human pandemic influenza viruses acquired their PB1 segment from avian viruses [70]. Early isolates of all these strains typically had multiple residues in the cytotoxic and inflammatory motifs. Over time, as the strains circulated in humans, these virulent residues became uncommon, not only because of the increased frequency of truncated PB1-F2 but also because the virulent residues mutated to less virulent sequences [59]. A similar trend has occurred in some other domestic species; however, whether because some species have inherited PB1 gene segments from avian viruses more recently (e.g., canine H3N2 IAV were acquired from avian viruses within the past 18 years), or because selection against the virulent residues has been less intense, influenza viruses of swine, dogs, and horses, as well as avian influenza viruses, are more likely to contain such residues. For example, although H1N2 and H3N2 SIVs rarely contained 3 or more of the four residues in the inflammatory motif, about 44% of H1N1 SIVs with non-truncated PB1-F2 had at least 3 such residues [68]; 83% of equine H3N8 had at least 3 inflammatory residues; and all canine H3N2 viruses had three [68].

Thus, influenza viruses of domestic mammals, as well as avian influenza viruses, may act as a reservoir of PB1-F2 with virulence-associated sequences.

## 7. Conclusions and Outstanding Questions

In spite of over 15 years’ worth of research on PB1-F2, many questions remain about the role and importance of the protein. The current knowledge of protein functioning suggests that PB1-F2 becomes a virulence factor for mammals when it is expressed at a high level as a full-length protein (or possibly as a C-terminal fragment) that contains specific virulence-associated residues within the C-terminus. All of these features depend on the specific protein sequence. 

At the molecular level, there is convincing evidence that PB1-F2 can cause cell death and inhibit the early IFN response, especially IFN-β. Other experiments suggest that it can also increase pro-inflammatory cytokines, and the increased inflammatory and pulmonary infiltration of leukocytes that is often observed in animals infected with virulence-associated variants of PB1-F2 suggests that the pro-inflammatory effect may be important in infections. However, it is still not clear what benefit the virus might obtain through increasing rather than inhibiting inflammation. The observation that PB1-F2 in avian influenza viruses may regulate immune activation and subsequent inflammatory response in chickens [10] may provide an explanation. Perhaps the protein is a true immune evasion molecule in birds, which are the natural host of influenza viruses, but acts inefficiently or inappropriately when infecting mammals. This might also explain the strong tendency for PB1-F2 proteins to lose virulence-associated residues through truncation and/or mutation as they evolve in mammalian hosts.

The recent identification of specific sequences associated with virulence (Section 5.2) may help resolve some of the variability seen in experiments using PB1-F2 from different viruses. Similarly, the fact that variant PB1-F2 proteins can be expressed even when stop codons, or a missing start ATG, prevent expression of the full-length protein may clarify some puzzling observations [4,5,19].

In spite of the occasionally confusing findings, it is clear that PB1-F2 can play a significant role in determining the virulence of influenza viruses. Although most human influenza viruses now contain PB1-F2 sequences that are expected to correlate with low virulence, there still exists a huge reservoir of influenza genomes in domestic animals that are capable of donating high-virulence PB1-F2 sequences, and mutations can restore virulence-associated sequences in human strains. PB1-F2 should therefore be monitored closely during surveillance, in order to help predict disease severity in upcoming influenza seasons.

## Figures and Tables

**Table 1:** Common length and virulent sequence variants of IAV PB1-F2.

Host	IAV Subtype	% of Isolates with Full Length Protein (≥90aa)	Common Length Variants (% of Isolates)	Common Virulent Residues in Isolates Expressing PB1-F2 ≥62aa
Human	1918pdmH1N1	100	N/A	Complete inflammatory motif with 66S and 70V
	Seasonal H1N1	<1	57aa (96.8)	
	2009pdmH1N1	0	11aa (100)	
	H1N1 SOIV ^a^	57	11aa (14.3) 57aa (14.3) 79aa (14.3)	100% have 2 inflammatory residues (62L and 82L).
	H1N2	100	N/A	No significant prevalence
	H1N2 SOIV ^a^	50	11aa (25)	100% have 2 inflammatory residues (62L and 82L).
	H2N2	>98	N/A	Majority have complete inflammatory motif.
	Seasonal H3N2	91.3	34aa (6.7)	Majority have 1 cytotoxic residue (70V). 10% of recent (2015–2017) isolates have 70V and 79R.
	H3N2 SOIV ^a^	>99	N/A	Majority have 2 inflammatory residues (62L and 82L)
	H5N1 ^b^	94	23aa (3)	Majority have complete inflammatory motif.
	H5N6 ^b^	66	57aa (27)	50% have complete inflammatory motif.
	H7N9 ^b^	86	57aa (5)	Majority have complete inflammatory motif.
Swine	H1N1	42	11aa (29), 79aa (19)	28.6% have complete inflammatory motif.
	H1N2	82	79aa (5.7), 57aa (5)	6.4% have complete inflammatory motif. Majority have 2 inflammatory residues (62L and 82L)
	H3N2	79	79aa (12), 35aa (3)	2.4% have complete inflammatory motif. Majority have 2 inflammatory residues (62L and 82L)
Equine	H3N8	50	81aa (50)	85% have 3 inflammatory residues.
	H7N7	75	34aa (25)	100% have 3 inflammatory residues.
Canine	H3N2	94.7	12aa (5.3)	100% have 3 inflammatory residues.
	H3N8	65.1	24aa (34.9)	22% have complete inflammatory motif.
Avian	H5N1	88.3	57aa (7.6), 24/25aa (2.03)	Majority have complete inflammatory motif.
	H7N9	83	25aa (6), 34aa (5)	Majority have complete inflammatory motif.
	H9N2	81.6	79aa (12), 57 (6.4)	50% have complete inflammatory motif.

Data are derived from analyses of PB1 sequences retrieved from GISAID and IRD databases as described in (Alymova et al., 2017 [59]). ^a^ SOIV indicates swine origin influenza viruses isolated from human infections. ^b^ Avian influenza viruses infecting humans.
